# Supplementary material for: The DNA glycosylase NEIL2 is protective during SARS-CoV-2 infection
Source: Nat Commun. 2023 Dec 9;14:8169. doi: 10.1038/s41467-023-43938-0 (PMC10710473; doi:10.1038/s41467-023-43938-0)

Supplementary Materials:

- Supplementary Fig. 1-7
- Supplementary Table 1-3
- Material availability statement
- Uncropped images for Supplementary Figure 3a
- Uncropped images for Supplementary Fig. 4b
- Uncropped images for Supplementary Fig. 4c
- Uncropped images for Supplementary Fig. 4d

Supplementary Figures

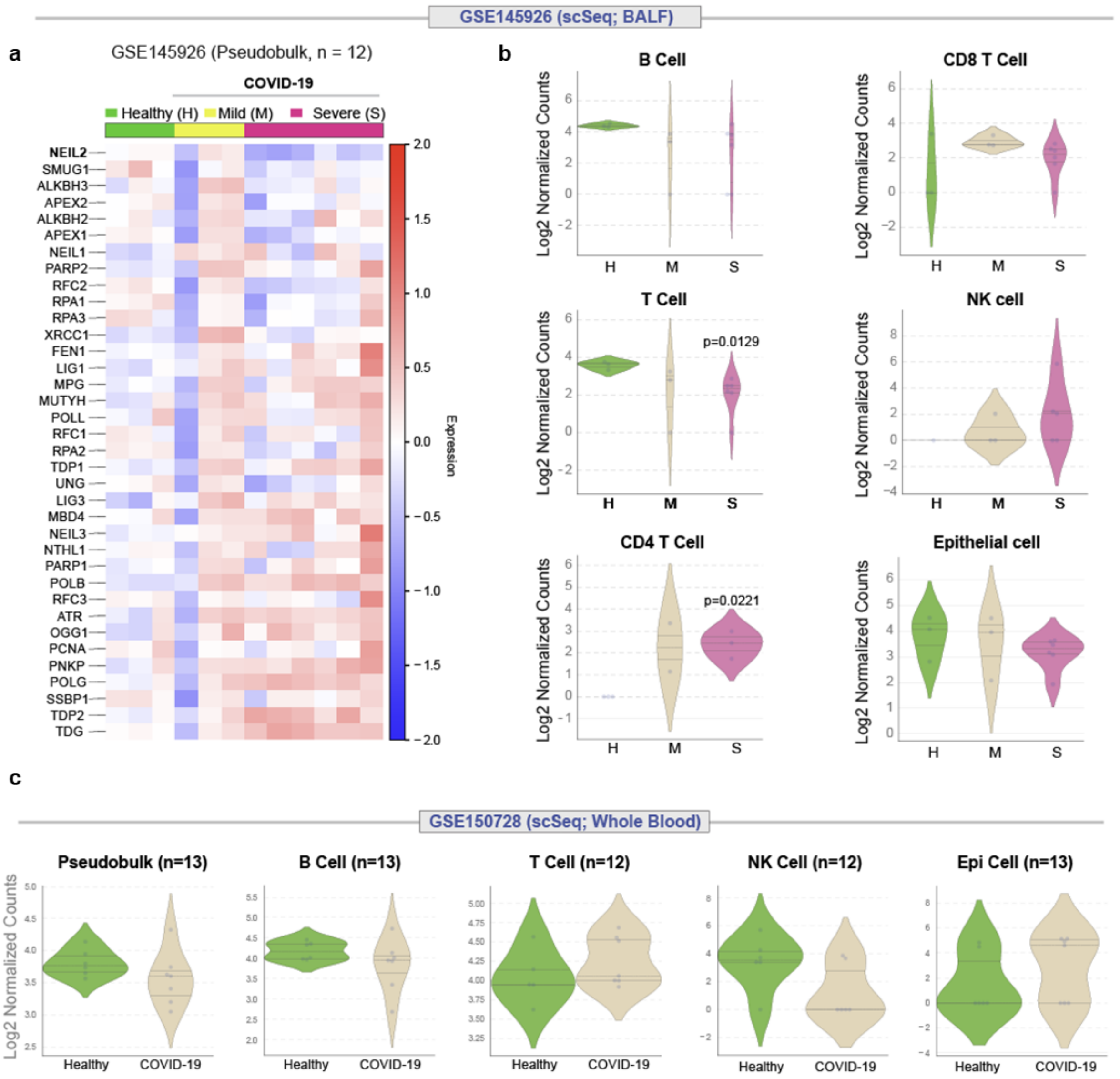

Supplementary Fig. 1. Downregulation of NEIL2 in COVID-19 patients

a) Heatmap showing changes in expression of genes that regulate DNA repair in bronchoalveolar lavage fluid (BALF) (GSE145926) obtained from healthy (H) and mild (M) or severe (S) COVID-19 patients. b, c) Swarm plots display the levels of expression of NEIL2 in various cell types in BALF (GSE145926) (b) and whole blood (GSE150728) (c). Data was analyzed using $\log_2$ (TPM+1) to compute the final log-reduced expression values for b and c (details in Methods).

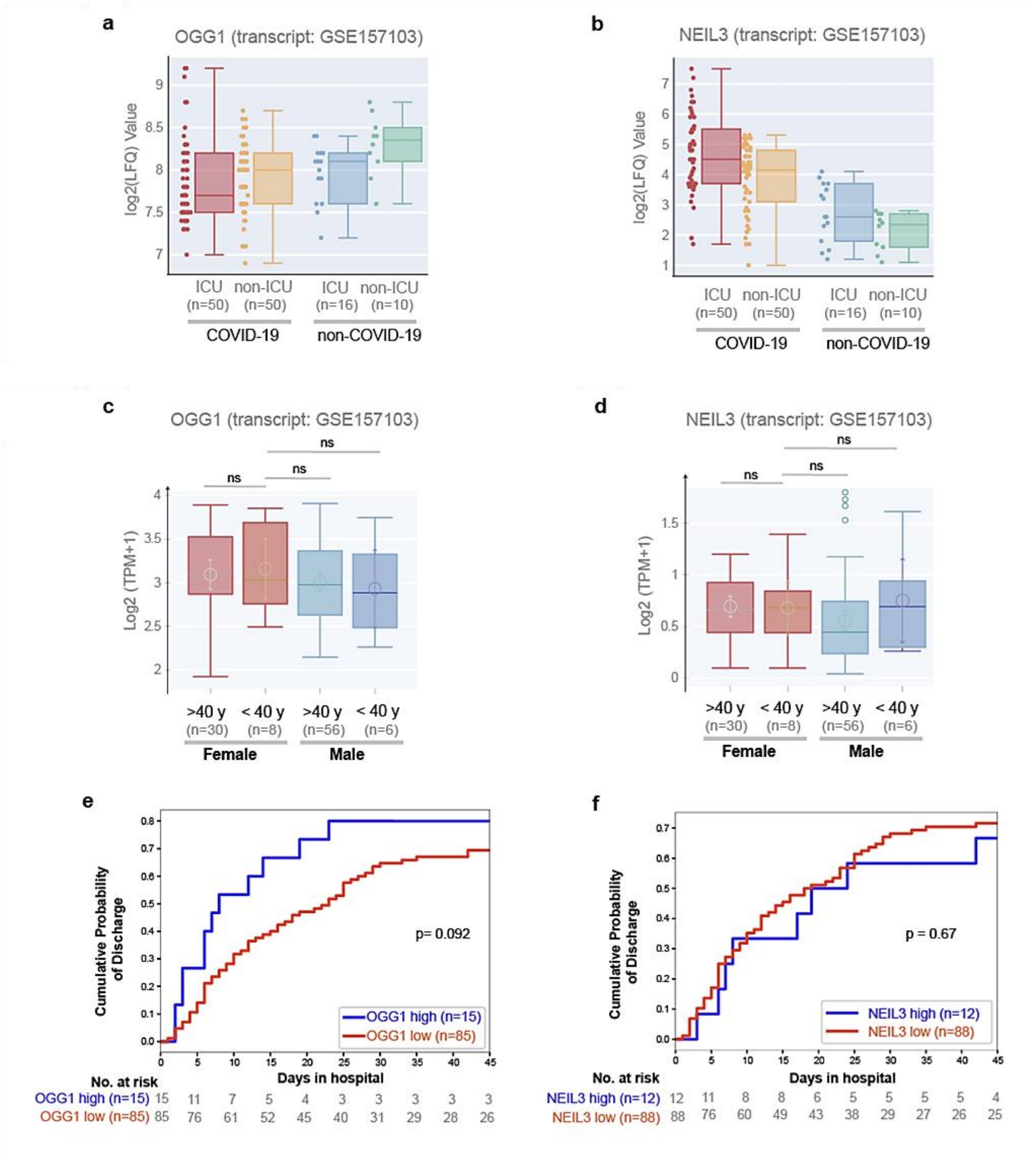

Supplementary Fig. 2. Correlation of OGG1 and NEIL3 expression levels with severity of COVID-19

a, b) Box plots display the levels of expression of OGG1 (a) and NEIL3 (b) in a cohort of hospitalized patients (GSE157103), stratified based on their level of care (Intensive Care Unit [ICU] vs. non-ICU) and diagnosis (COVID-19 vs. non-COVID-19). c, d) Whisker plots display the levels of expression of OGG1 (c) and NEIL3 (d) in groups of patients (GSE157103) stratified

by sex and age (using 40 years as a cut-off). All boxplots (a-d) have a horizontal line at the median and the box extends to the first and third quartile with whiskers extending to 1.5-times the interquartile range. e, f) Kaplan-Meier plots display the cumulative probability of discharge based on high vs. low levels of expression of OGG1 (e) and NEIL3 (f). Unpaired two-tailed Student's t test p-values to compare two groups was used for statistical analysis.

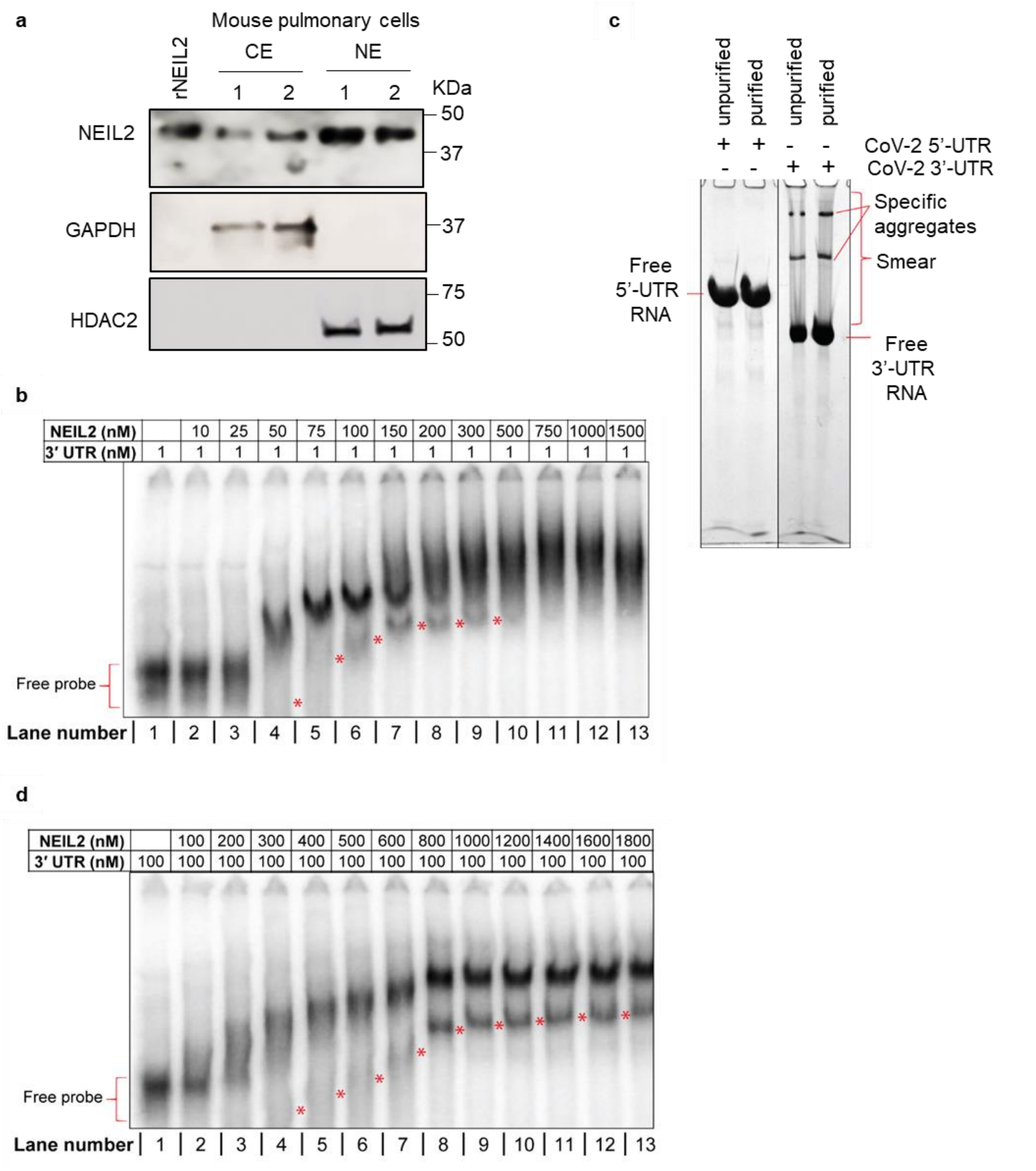

Supplementary Fig. 3. Analysis of binding of NEIL2 to SARS-CoV-2 3'-UTR by RNA-EMSA a) Immunoblot analysis of NEIL2 levels in cytosolic (CE) and nuclear (NE) extracts from mouse pulmonary cells. GAPDH and HDAC2 were used as cytosolic and nuclear loading controls, respectively. b) Titration of 1 nM radiolabeled 3'-UTR RNA with NEIL2 in an affinity-based binding study; '*' indicates complexes formed by upshifting of an aggregated faster migrating RNA species upon binding to NEIL2. (c) Crude *in vitro* transcription reaction (unpurified) and

RNA purified from the transcription reaction analyzed by urea PAGE showing the proneness of the 3’-UTR, but not the 5’-UTR RNA to aggregate. (d) Titration of 100 nM unlabeled 3’-UTR RNA, traced with radiolabeled RNA, with NEIL2 in a stoichiometry-based binding study. Representative images from n=3 independent experiments are shown for a-d.

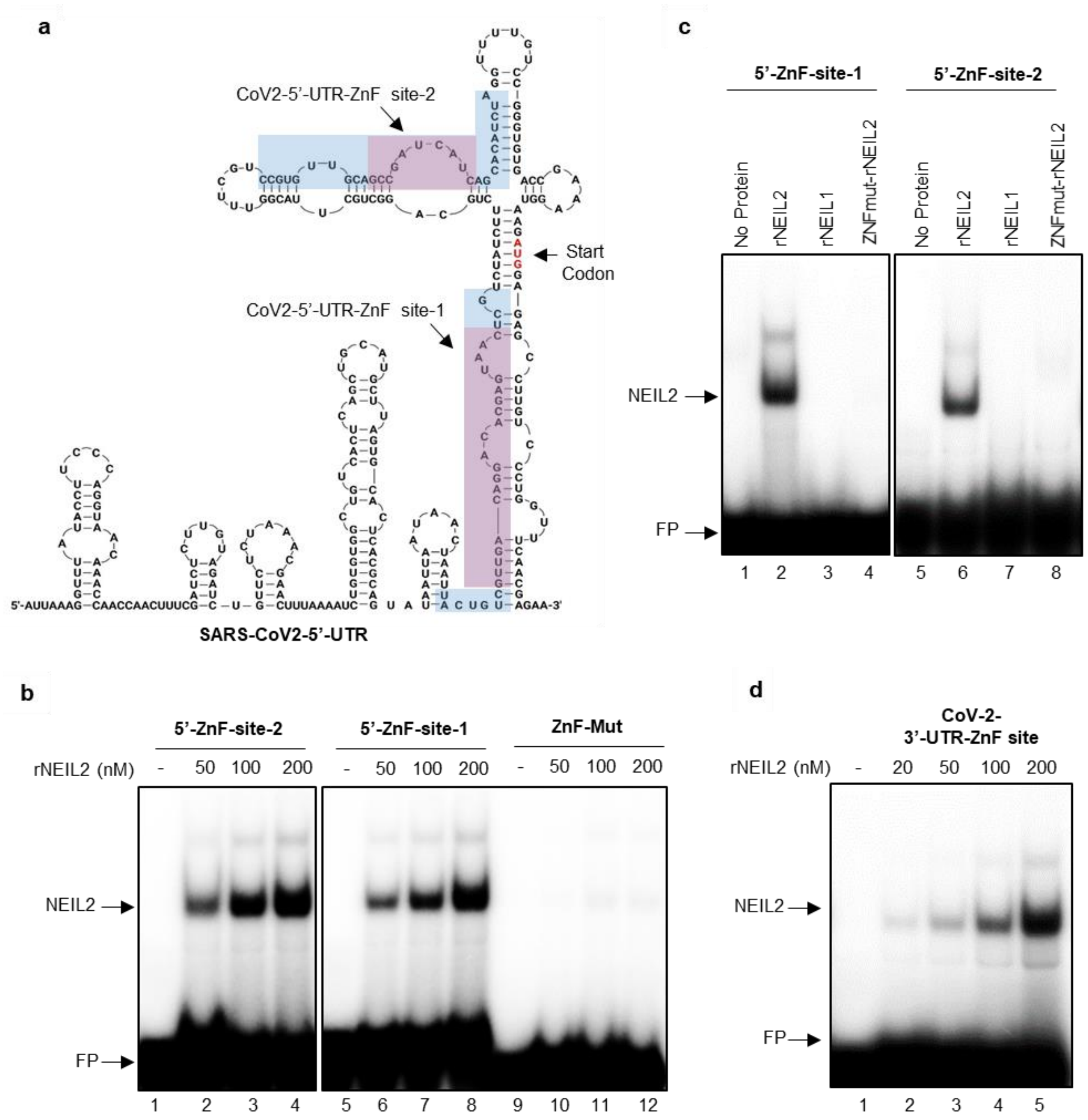

Supplementary Fig. 4. NEIL2 binds to ZnF sites located at 5'- and 3'-UTRs of SARS-CoV-2 genomic RNA

a) Schematic shows secondary structure of 297 nt long 5'-region of SARS-CoV-2 RNA as predicted by online tool available at http://rna.urmc.rochester.edu/RNAstructure.html and created with BioRender.com. The two zinc finger binding sites, 1 and 2, are shown in pink flanked by additional nucleotides (marked in blue) used in RNA-EMSA studies. b) RNA-EMSA showing the binding of rNEIL2 (50-200 nM) with ^{32}P-labeled ~38-mer RNA probe containing zinc finger (ZnF) binding sites (site-1, lanes 6-8 and site-2, lanes 2-4) derived from SARS-CoV-2-5'-UTR or a RNA probe devoid of ZnF site (ZnF-mut, lanes 10-12). c) RNA-EMSA of 200 nM rNEIL2, rNEIL1 or

ZnF-mutant rNEIL2 with ^{32}P-labeled RNA probe containing ZnF-site-1 and ZnF-site-2 derived from SARS-CoV-2-5'-UTR. d) RNA-EMSA showing the binding of rNEIL2 (20-200 nM) with ^{32}P-labeled RNA probe containing zinc finger protein binding site derived from SARS-CoV-2 3'-UTR. Representative images from n=3 independent experiments are shown for b-d. FP: free probe.

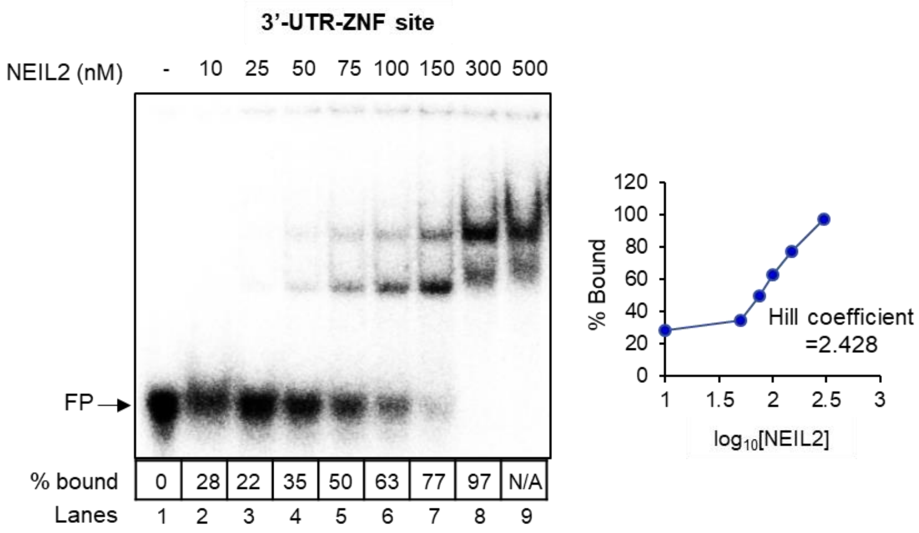

Supplementary Fig. 5. Analysis of binding of NEIL2 to CoV-2 3’-UTR ZnF site
Titration of 1 nM radiolabeled CoV-2 3’-UTR ZnF site containing RNA oligo with rNEIL2 in an affinity-based binding study as analyzed by RNA-EMSA. Dose-response curve generated by plotting the percentage of bound RNA, as shown in left panels, against log_{10} of rNEIL2 concentrations for calculation of Hill coefficient. Representative image and quantification from n=3 independent experiments is shown. FP: free probe.

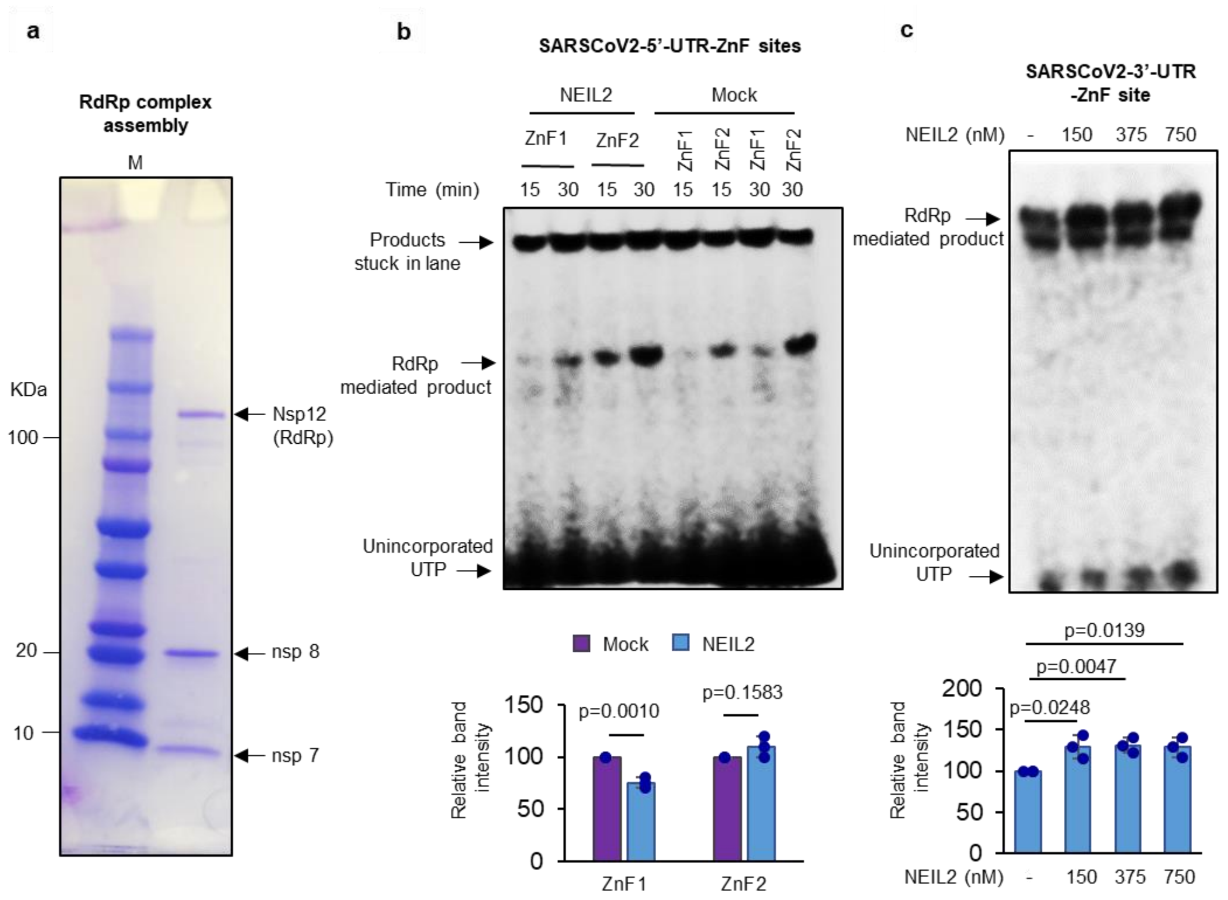

Supplementary Fig. 6. Effect of NEIL2 on SARS-CoV-2 RNA replication

a) The viral RNA-dependent RNA polymerase (RdRp) complex (nsp12-nsp7-nsp8 in 1:2:2 molar ratio) was assembled from individually purified recombinant proteins and run in SDS-PAGE following 3 h assembly reaction. M: Protein molecular weight marker. b, c) The RdRp assembly complex was pre-incubated with 150 nM of recombinant NEIL2 for the indicated amount of time (b) and with buffer only (-) or indicated amounts (c). The RNA directed RNA polymerase activity was assessed by radiolabeled UMP incorporation on individual RNA substrates containing ZnF sites derived from SARS-CoV-2 5'-UTR (b) and SARS-CoV-2 3'-UTR (c) primed with short RNA oligo. Representative images from 3 independent experiments are shown. Histograms show the relative radioactive incorporated band intensity (as a measure of RdRp activity) with mock buffer incubated sample arbitrarily taken as 100 for b. Error bars represent ± standard deviation from the mean; n=3 independent experiments. p-values (unpaired two-tailed Student's t test) vs. mock for b and; no NEIL2 group for c. Representative image from n=3 independent experiments is shown for a-c.

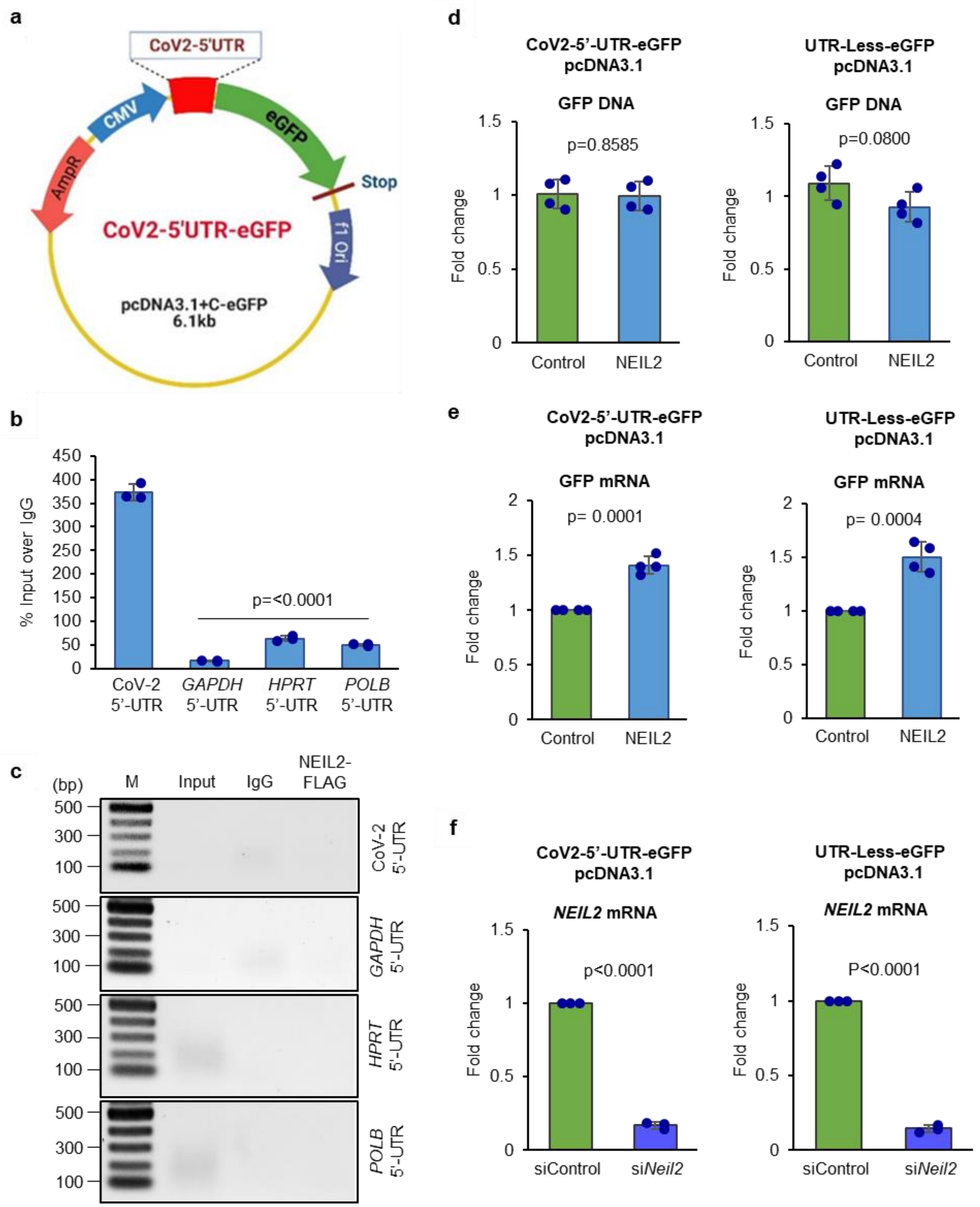

Supplementary Fig. 7. Transfection of the reporter plasmid (CoV2-5'-UTR-eGFP) in cultured cells

a) 5'-region of SARS-CoV-2 genome (297 nt) including 5'-UTR was cloned upstream of Green Fluorescence Protein (eGFP) in mammalian expression plasmid under the control of CMV promoter as shown in the schematic. b, c) Human BEAS-2B cells stably expressing NEIL2-FLAG protein were transfected with CoV2-5'-UTR-eGFP construct and their cell extracts subjected to RNA-ChIP analysis using the anti-FLAG or -IgG antibodies. Total RNA was isolated from eluates

and subjected to cDNA preparation in presence (b) and absence (c) of reverse transcriptase, and analyzed by RT-qPCR and PCR with 5'-UTR region specific primers for CoV-2, *GAPDH*, *HPRT*, and *POLB* mRNAs, respectively. Results are represented as % inputs over IgG where error bars show ±standard deviation from the mean, n=3 independent experiments (b). Representative images (n=3) of PCR run without reverse transcriptase for indicated gene regions are shown (c). d, e) Human BEAS-2B cells, control or stably expressing NEIL2-FLAG protein (NEIL2) were transfected with CoV2-5'-UTR-eGFP construct (left panels) or a plasmid devoid of CoV-2-5'-UTR (UTR-Less-eGFP) (right panels) for 16 h and GFP genomic DNA (d) and GFP mRNA (e) levels were analyzed by qPCR and RT-qPCR, respectively; n=4 independent experiments. f) HEK293 cells were transfected with control or -NEIL2 specific siRNA for 48 h and *NEIL2* mRNA expression was analyzed by RT-qPCR (n=3 independent experiments). All error bars represent ±standard deviation from the mean and p-values (unpaired two-tailed Student's t test) vs. CoV2-CoV2-5'-UTR-eGFP transfected group for b, control for d and e, siControl group for f.

Supplementary Table 1

List of specific primers used in the study.

Primers	Sequence (5'→3')	Species	Assay
HamPOLA1-LA-F	CGCTGATTGGCTTTGGTACTG	Hamster	LA-qPCR
HamPOLA1-LA-R	GACACCTGTTCAGTGTTGGAC	Hamster	LA-qPCR
HamRNAPII-LA-F	AAGGGGGAGAGACAAACTGC	Hamster	LA-qPCR
HamRNAPII-LA-R	TGTGGAGTCAGGCATGGAAC	Hamster	LA-qPCR
HamPOLA1-SA-F	CACCCAGCTGTGCTTTTACC	Hamster	SA-PCR
HamPOLA1-SA-R	TCCCAGCATAACTGGCGAAG	Hamster	SA-PCR
HamRNAPII-SA-F	GAGATGTCTTCCTGGAGCGG	Hamster	SA-PCR
HamRNAPII-SA-R	GCTTGTAAGGGCCACTGTCT	Hamster	SA-PCR
h*GAPDH*-mRNA-F	CATCACTGCCACCCAGAAGA	Human	RT-qPCR
h*GAPDH*-mRNA-R	TTCTAGACGGCAGGTCAGGT	Human	RT-qPCR
h*NEIL2*-mRNA-For2	CCTGTCTGCTATACACTGC	Human	RT-qPCR
h*NEIL2*-mRNA-Rev3	CCTGCAGCCAGGCTGTACTG	Human	RT-qPCR
h*NEIL1*-mRNA-F	CGCTCCTCCCCAAAACTAGG	Human	RT-qPCR
h*NEIL1*-mRNA-R	CTGCTTTGCCACTTGCCTCT	Human	RT-qPCR
18S-rRNA-Fov	GTAACCCGTTGAACCCCATT	Human	RT-qPCR
18S-rRNA-Rev	CCATCCAATCGGTAGTAGCG	Human	RT-qPCR
h*CCL2*-mRNA-F	AGCAGCAAGTGTCCCAAAGA	Human	RT-qPCR
h*CCL2*-mRNA-R	TTGGGTTTGCTTGTCCAGGT	Human	RT-qPCR
h*CCL3*-mRNA-F	GCTCTCTGCAACCAGTTCTCT	Human	RT-qPCR
h*CCL3*-mRNA-R	GGCTTCGCTTGGTTAGGAAGA	Human	RT-qPCR
h*CXCL10*-mRNA-F	ACTGTACGCTGTACCTGCAT	Human	RT-qPCR
h*CXCL10*-mRNA-R	TGATGGCCTTCGATTCTGGAT	Human	RT-qPCR
h*TNFα*-mRNA-For	TCCTCTCTGCCATCAAGAGC	Human	RT-qPCR
h*TNFα*-mRNA-Rev	TCCTCACAGGGCAATGATCC	Human	RT-qPCR
h*IL6*-5'UTR-For	CCCACCGGGAACGAAAGAG	Human	RT-qPCR
h*IL6*-5'UTR-Rev	AGAAGGCAACTGGACCGAAG	Human	RT-qPCR
h*IL1β*-mRNA-F	CCTGAGCTCGCCAGTGAAAT	Human	RT-qPCR
h*IL1β*-mRNA-R	TCCATGGCCACAACAACTGA	Human	RT-qPCR
h*GAPDH*-5'UTR-F	CCTCCCGCTTCGCTCTCT	Human	RNA-ChIP-qPCR
h*GAPDH*-5'UTR-R	CATGGTGTCTGAGCGATGTG	Human	RNA-ChIP-qPCR
h*HPRT*-5'UTR-F	CCTCTTGCTGCGCCTCC	Human	RNA-ChIP-qPCR
h*HPRT*-5'UTR-R	GGGTCGCCATAACGGAGC	Human	RNA-ChIP-qPCR
h*POLB*-5'UTR-F	CTCCTGCTCCCGTCTCCAAG	Human	RNA-ChIP-qPCR
h*POLB*-5'UTR-R	CCCCAGAGTGTTCAGAACCAG	Human	RNA-ChIP-qPCR

CoV2-5'UTRznf-For	TGTCGTTGACAGGACACGAG	SARS-CoV-2	RNA-ChIP-qPCR
CoV2-5'UTRznf-Rev	TTACCTTTCGGTCACACCCG	SARS-CoV-2	RNA-ChIP-qPCR
CoV2-3'UTRznf-For	CAATGCTAGGGAGAGCTGC	SARS-CoV-2	RNA-ChIP-qPCR
CoV2-3'UTRznf-Rev	AGAAGCTATTAAAATCACATGGGGA	SARS-CoV-2	RNA-ChIP-qPCR
CoV2-gene-For2	ACTTTAATGTTTTATTCTCTACAGT	SARS-CoV-2	RNA-ChIP-qPCR
CoV2-gene-Rev2	ACAAATATTTTTCTCACTAGTGGTC	SARS-CoV-2	RNA-ChIP-qPCR
eGFP-For	GGCAAGCTGACCCTGAAGTT	-	RT-qPCR
eGFP-Rev	CTTGTAGTTGCCGTCGTCCT	-	RT-qPCR

Supplementary Table 2

Sequences of *in vitro* transcribed SARS-CoV-2 UTRs used in RNA-EMSA

**Label**	***in vitro* transcribed CoV-2 untranslated regions (5'→3')**
Full-length CoV-2 5'-UTR	5'AUUAAAGGUUUAUACCUUCCCAGGUAACAAACCAACCAACUUUCGA UCUCUUGUAGAUCUGUUCUCUAAACGAACUUUAAAAUCUGUGUGGCU GUCACUCGGCUGCAUGCUUAGUGCACUCACGCAGUAUAAUUAAUAAC UAAUUACUGUCGUUGACAGGACACGAGUAACUCGUCUAUCUUCUGCA GGCUGCUUACGGUUUCGUCCGUGUUGCAGCCGAUCAUCAGCACAUCU AGGUUUUGUCCGGGUGUGACCGAAAGGUAAGAUGGAGAGCCUUGUCC CUGGUUUCAACGAGAA 3'
Full-length CoV-2 3'-UTR	5'CAAUCUUUAAUCAGUGUGUAACAUUAGGGAGGACUUGAAAGAGCCA CCACAUUUUCACCGAGGCCACGCGGAGUACGAUCGAGUGUACAGUGA ACAAUGCUAGGGAGAGCUGCCUAUAUGGAAGAGCCCUAAUGUGUAAA AUUAAUUUUAGUAGUGCUAUCCCCAUGUGAUUUUAAUAGCUUCUUAG GAGAAUGACAAAA 3'

Supplementary Table 3

List of RNA oligos used in RNA-EMSA and *in vitro* RdRp assay (DNA bases are shown in blue).

RNA Oligo label	Oligo sequence (5'→3')	Assay
CoV-2 5'-UTR-ZnF site- 1	TAATrUrArCrUrGrUrCrGrUrUrGrArCrArGrGrArCrArCrGrArGrUrArArCrUrCrGTCTA	RNA-EMSA
CoV-2 5'-UTR-ZnF site- 2	TCGTrCrCrGrUrGrUrUrGrCrArGrCrCrGrArUrCrArUrCrArGrCrArCrArUrCrUrAGGTT	RNA-EMSA
Nonspecific Control-ZnF-Mut	TTAGrCrArUrArGrUrArCrArUrCrCrCrCrUrGrArGrCrArCrArCrGGCAC	RNA-EMSA
CoV-2 3'-UTR-ZnF site	Phos-rUrCrCrCrCrArUrGrUrGrArUrUrUrUrArArUrArGrCrUrUrCrUrUrArGrGrArGrArArUrGrArCrA	RdRp assay
CoV-2 5'-UTR ZnF-site-1-RdRp	Phos-rUrGrUrCrGrUrUrGrArCrArGrGrArCrArCrGrArGrUrArArCrUrCrGrCrGrUrArGrUrUrUrUrCrUrArCrGrCrG	RdRp assay
CoV-2 5'-UTR ZnF site-2-RdRp	Phos-rTrGrTrTrGrCrArGrCrCrGrArTrCrArTrCrArGrCrArCrArTrCrCrGrCrGrUrArGrUrUrUrUrCrUrArCrGrCrG	RdRp assay
CoV-2 3'-UTR-short primer-R	rUrGrUrCrArU	RdRp assay

Material availability statement: All the material used in the study is available from authors on request.

Uncropped images for Supplementary Figure 3a

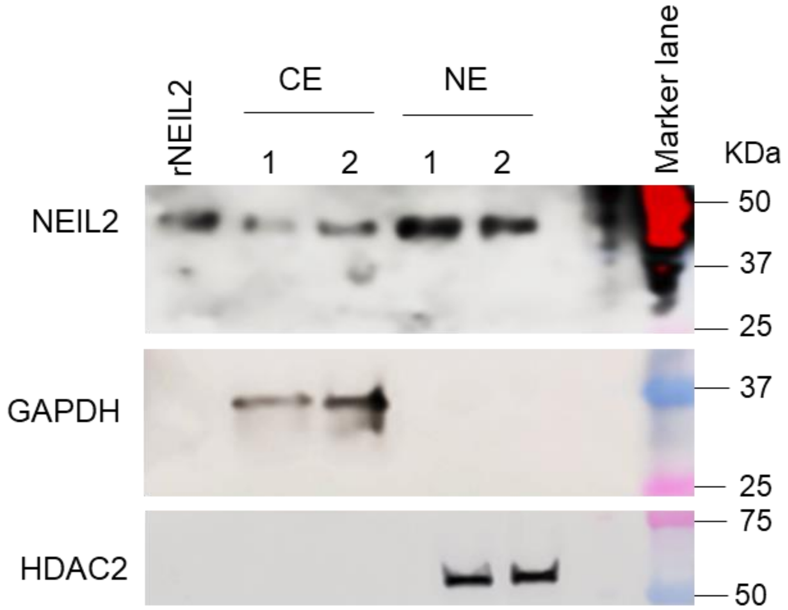

Uncropped images for Supplementary Figure 4b

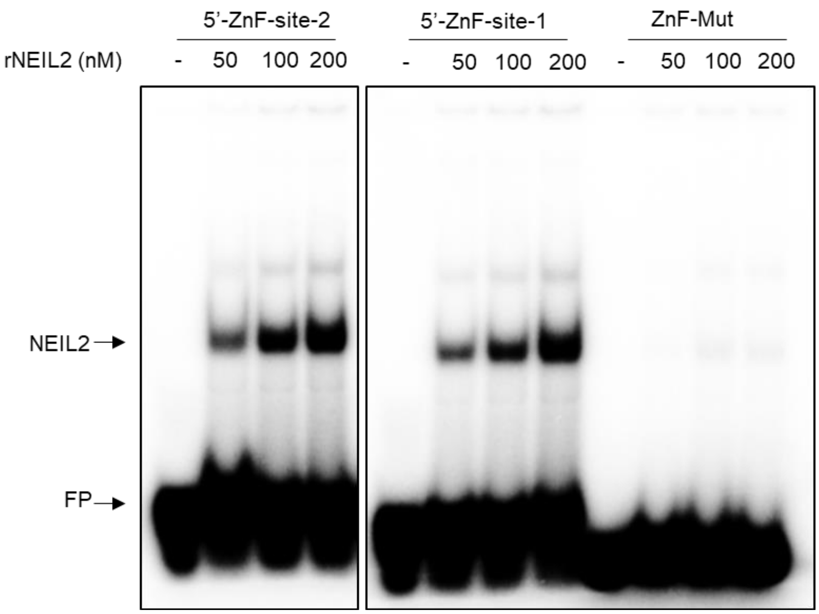

Uncropped images for Supplementary Figure 4c

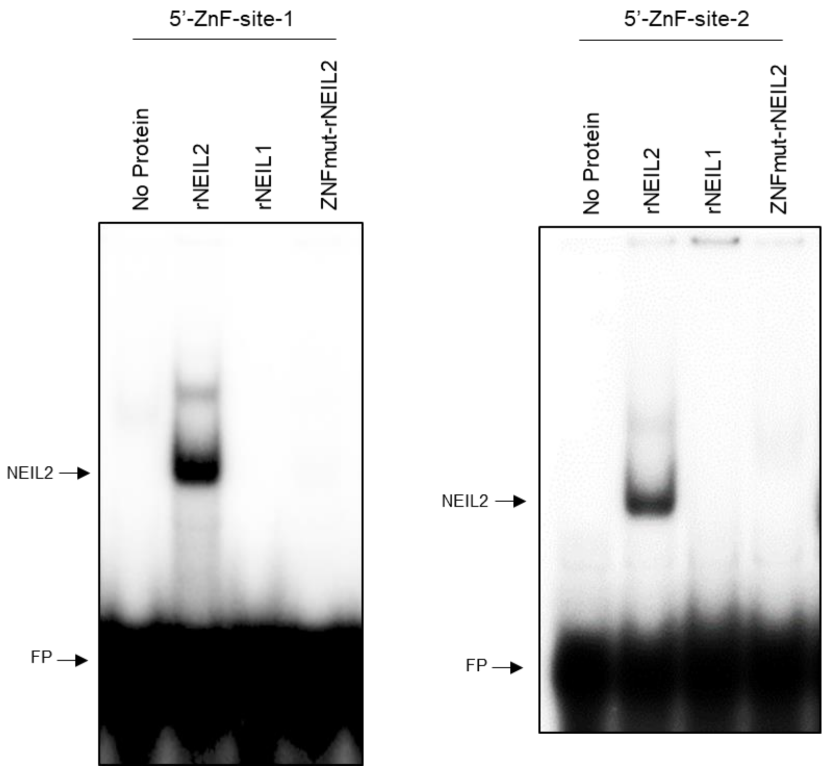

Uncropped image for Supplementary Figure 4d

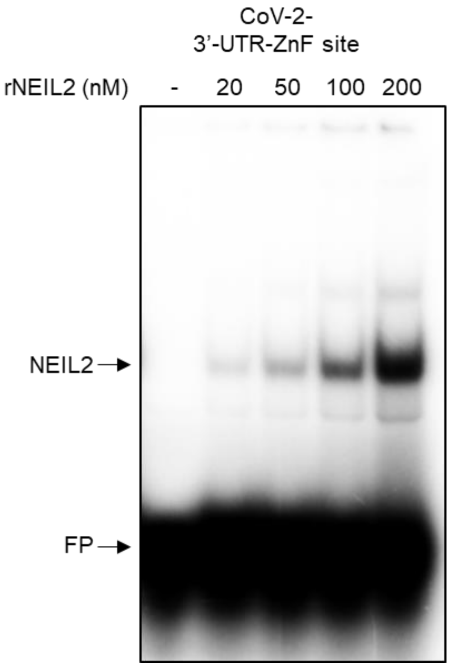

Supplement: Supplementary file 1 — Supplementary Information [file 41467_2023_43938_MOESM1_ESM.pdf]
